# Evaluation of Predictive Risk Factors of Persistent Hypertension in Hyperaldosteronism After Surgery

**DOI:** 10.5812/ijem-156728

**Published:** 2025-04-30

**Authors:** Amal Ourdi, Youssra Laalaoua, Imane Assarrar, Bouichrat Nisrine, Siham Rouf, Hanane Latrech

**Affiliations:** 1 Department of Endocrinology-Diabetology and Nutrition, Mohammed VI University Hospital Center, Faculty of Medicine and Pharmacy, University of Mohammed 1st, Oujda, Morocco; 2 Laboratory of Epidemiology, Clinical Research and Public Health, Mohammed VI University Hospital Center, Faculty of Medicine and Pharmacy, University of Mohammed 1st, Oujda, Morocco

**Keywords:** Primary Hyperaldosteronism, Endocrine Hypertension, Conn’s Adenoma, Adrenal Gland, Persistent Hypertension, Predictive Factors

## Abstract

**Background:**

Primary hyperaldosteronism (PHA) is a common cause of secondary arterial hypertension (AH), characterized by autonomous aldosterone secretion. It is frequently underdiagnosed and may persist even after surgical intervention.

**Objectives:**

The present study aimed to identify preoperative factors that could predict whether hypertension would persist or normalize following surgery and to outline relevant diagnostic characteristics.

**Methods:**

We conducted a descriptive, analytic, retrospective cohort study at a single center. The study included patients with PHA who were followed up at the Department of Endocrinology, Diabetology, and Nutrition in a hospital affiliated with Mohamed the First University of Oujda (CERBO), admitted between December 2014 and August 2023. Data were retrospectively collected from patient records over a 9-year period, involving 27 patients with PHA confirmed by an elevated aldosterone-to-renin ratio (ARR). Persistent disease was defined by persistent hypokalemia and hypertension (blood pressure > 140/90 mm Hg) after six months. Patients were divided into two groups: Those with complete resolution of hypertension (group A) and those with persistent hypertension (group B). Data were analyzed using SPSS version 21.

**Results:**

The mean age of patients was 48.47 ± 10.87 years, with a female predominance (66.7%). The etiological assessment identified Conn’s adenoma in 70.4% (n = 19) of cases and bilateral adrenal hyperplasia in 29.6% (n = 8). Surgery was performed in 51.9% (n = 14) of cases, with 50% (n = 7) maintaining persistent hypertension post-surgery, while 28.6% (n = 4) showed a reduction in antihypertensive medications. Two predictive factors for persistent hypertension were identified: Age > 50 years and hypertension duration > 5 years. Predictive factors for normalization of hypertension post-surgery included systolic blood pressure (SBP) < 140 mm Hg, diastolic blood pressure (DBP) < 90 mm Hg, glomerular filtration rate (GFR) > 90 mL/min/1.75 m^2^, and a low incidence of diabetes and dyslipidemia.

**Conclusions:**

This study demonstrates that PHA can lead to resistant hypertension, highlighting the necessity for further research in this area.

## 1. Background

Primary hyperaldosteronism (PHA), also known as Conn’s syndrome (1), is characterized by a benign adrenocortical mass that secretes aldosterone in a largely autonomous manner. This condition often leads to severe hypertension and hypokalemia (2). Previously thought to account for less than 1% of hypertensive cases, recent studies have shown a significantly higher prevalence, ranging from 5% to 20% in various reports (3-6). Despite this, PHA remains underdiagnosed and is considered a relatively rare disease. The most common cause of PHA is Conn’s adenoma, which is associated with a high rate of cardiovascular morbidity, increasing the risk of cardiovascular disease, atrial fibrillation, and stroke. Accurate diagnosis of PHA can significantly improve patient outcomes by reducing blood pressure, optimizing treatment regimens, and decreasing overall cardiovascular risk (7). The predictive risk factors for persistent hypertension following adrenal adenoma surgery are not fully understood. Therefore, this study aims to evaluate clinical indicators, therapeutic approaches, and outcomes of PHA, with a focus on identifying predictors of successful outcomes and persistent hypertension post-surgery.

## 2. Objectives

The primary outcome of this study was to identify preoperative factors that could predict the persistence or normalization of hypertension following surgery. These factors included gender, age, duration of hypertension, the number of antihypertensive medications, Body Mass Index (BMI), systolic blood pressure (SBP), diastolic blood pressure (DBP), glomerular filtration rate (GFR), and the presence of diabetes and dyslipidemia. The secondary outcome was to describe the diagnostic characteristics of patients suspected of having PHA.

## 3. Methods

### 3.1. Study Design

This was a descriptive, analytical, retrospective cohort and monocentric study of patients with PHA followed up at the Department of Endocrinology, Diabetology, and Nutrition in a hospital affiliated with Mohamed the First University of Oujda (CERBO), Oujda, Morocco. All included patients were admitted between December 2014 and August 2023. The patients gave their oral consent to use their medical data. The local ethical committee of biomedical research approved the study protocol.

### 3.2. Study Population

Diagnosis is often based on screening; our selection criteria were:

- Grade 3 hypertension

- Diastolic blood pressure (DBP ≥ 110 mmHg)

- Resistant hypertension (SBP ≥ 140 mmHg and/or DBP ≥ 90 mmHg despite triple antihypertensive therapy including an optimal-dose thiazide diuretic)

- Hypertension and hypokalemia

- Adrenal incidentaloma with hypertension and/or hypokalemia

- Hypertension with disproportionate impact on target organs

- Hypertension in young people

- Hypertension with a family history of early-onset hypertension or stroke before the age of 40

- Hypertension with a family history of PHA in the first degree

### 3.3. Inclusion and Exclusion Criteria

**Figure 1.**
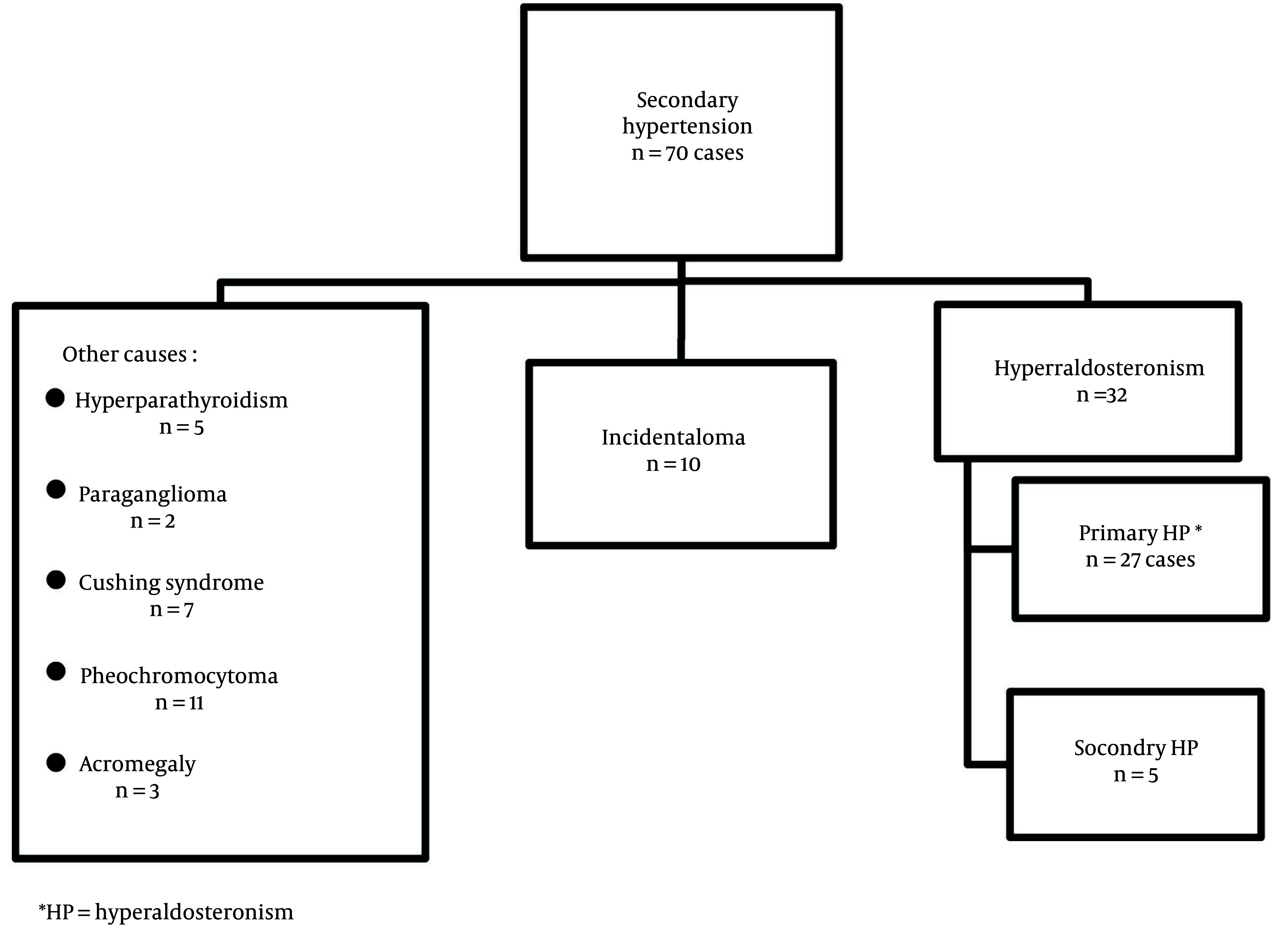
Study population

**Figure 2.**
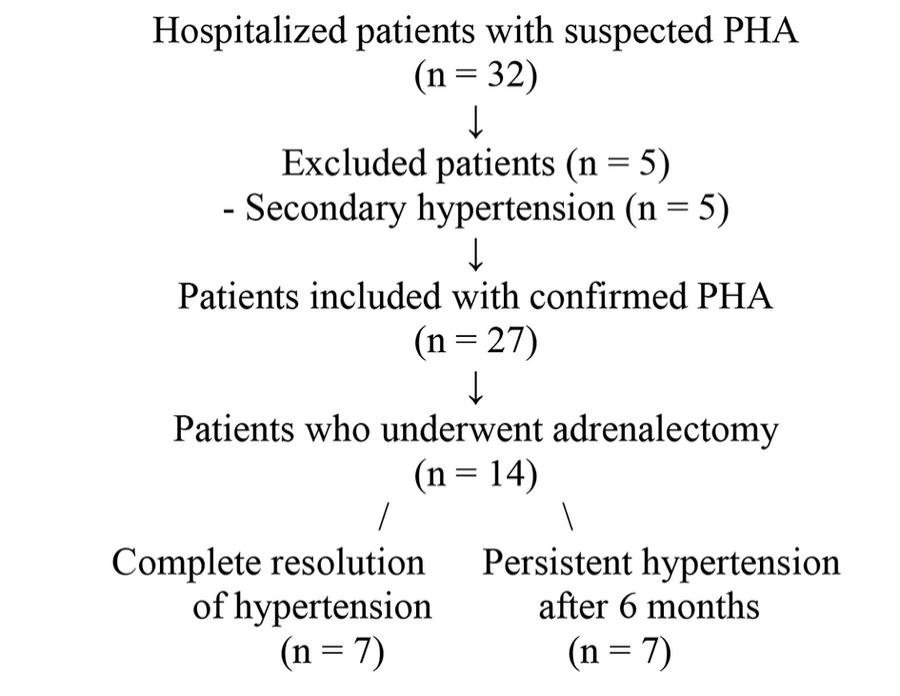
Flowchart of patient selection and outcomes

Our inclusion criteria included patients aged from 18 to 75 years diagnosed with PHA and complete medical records. Our exclusion criteria were patients with secondary hyperaldosteronism, essential hypertension, endocrine hypertension other than PHA, and hypertensive patients with non-functional incidentalomas. Due to the rarity of the condition and the monocentric nature of the study, only 27 confirmed PHA cases were included (Figures 1 and 2). 

### 3.4. Definitions

Demographic and clinical data were collected from the patients’ medical records, including:

- Clinical and socio-demographic data: Age (years), sex, BMI (kg/m^2^), waist circumference (cm), SBP (mmHg), DBP (mmHg), stage of hypertension, personal background, circumstances of discovery, antihypertensive treatment, family history (stroke, hypertension), and diabetes history. Hypertension was defined as blood pressure above 140/90 mm Hg, taken over three successive days with manual intake.

- Blood workup data: Serum potassium and sodium, urine measurement of Na⁺ and K⁺ in 24-hour, plasma aldosterone, plasma renin, and aldosterone/renin plasma ratio. Normal laboratory values for aldosterone dosage are: Standing position, 61.3 to 979 pmol/L; supine position, 32.5 to 655 pmol/L. Normal laboratory values for potassium assay are 3.5 - 5 mmol/L. The assays were performed using a validated automated Chemiluminescence Immunoassay System. We used the aldosterone/renin ratio (ARR) because it is more sensitive and has lower variability than other assays (8).

- Morphological evaluation: Performed using abdominal CT with the adrenal protocol.

- Evaluation of impact and complications: Included metabolic profile (lipid profile, HbA1c), serum creatinine, electrocardiogram, echocardiography, and 24-hour proteinuria.

### 3.5. Statistical Analysis

We used SPSS version 21. Univariate and multivariate logistic regression analyses were conducted. Confounding factors were controlled in the regression model. P-values < 0.05 were considered statistically significant.

## 4. Results

The mean age at diagnosis was 48 ± 10.87 years, with a female-to-male ratio of 2. Table 1 shows the characteristics of the studied population (Table 1). Upright posture plasma aldosterone concentration (PAC) was elevated in all cases, with a mean of 1555.81 ± 1396.41 pmol/L, and supine posture PAC averaged 729.29 ± 657.60 pmol/L. The mean upright posture direct renin (DR) was 11.58 ± 8.62 mIU/L and 8.67 ± 8.53 mIU/L in the supine posture. The ARR measured under upright and supine posture was high in all cases, 129.07 ± 61.90 and 88.81 ± 27.76, respectively.

**Table 1.** Circumstances of Primary Hyperaldosteronism Discovery and General Characteristics of the Studied Population

Variables	Values ^a^
**Age at diagnosis (y)**	48 ± 10.8
**Hypertension, onset age (y)**	43 ± 10
**Circumstances of PHA discovery**	
Severe and resistant hypertension	37
Incidental discovery	22
Routine screening	7
Complications of hypertension	16
**Main mode of PHA presentation**	
High BP and hypokalemia	66.6
**Family history**	
Hypertension	40.7
Coronary heart disease	3.70
Diabetes	33.3
Dyslipidemia	11.1
**Waist circumference (cm)**	97.7 ± 10.1
**BMI (kg/m** ^**2**^ **)**	28.7 ± 3.6
**Overweight and obesity**	
Overweight	58.1
Obesity grade	37
**Diabetes**	37
**HbA1C**	6.05 ± 1.35
**Dyslipidemia**	37
**24-hour ambulatory blood pressure monitoring found hypertension**	51.9
**Impact of hypertension**	
LVH	29.6
Hypertensive nephropathy	11.1
Hypertensive retinopathy	7.40
Stroke	11.1
**Positive 24-hour urine protein test**	14.8
**Microalbuminuria**	11.1
**Treatment of hypertension at diagnosis**	
No treatment	37.4
Monotherapy	29.6
Bi-therapy	15
Tri-therapy	18

Abbreviations: PHA, primary hyperaldosteronism; BMI, Body Mass Index; LVH, left ventricular hypertrophy.

^a^ Values are presented as Mean ± SD or %.

Computed tomography (CT) was normal in 25.9% of cases (n = 7), revealed adrenal adenoma in 70.4% (n = 19), and bilateral adrenal adenoma in 3.7% (n = 1). Since some patients with PHA having hyperplasia may show normal CT, we have added them to patients with adrenal hyperplasia (Table 2). 

**Table 2.** Distribution of Etiologies According to the Different Series of Literature

Author, Country, Year (Ref.)	Cases (No.)	Etiology		
		Adenoma (%)	Adrenal Bilateral (%)	Hyperplasia Unilateral (%)
**Present study**	27	70	30	0
**Kilani et al., Tunisia, 2014 (** 9 **)**	25	72	16	12
**Haddam et al., Algeria, 2013 (** 10 **)**	20	75	25	0
**Cordoliani et al., France, 2017 (** 11 **)**	22	72	9	13

In the preoperative setting, blood pressure and hypokalemia should be optimally managed, preferably with a mineralocorticoid receptor antagonist, in accordance with the Endocrine Society guidelines. Typically, an initial dose of 12.5 mg to 25 mg of spironolactone is used, with titration up to a maximum dose of 100 to 200 mg. However, there are no specific recommendations regarding the optimal duration of preoperative treatment (12). We adopted spironolactone (100 mg/d) for 14.8% of our patients (n = 2).

Surgery was performed in 51.85% of cases (n = 14), with adenoma confirmed in 92.85% (n = 13) and hyperplasia in 7.14% (n = 1). The rest did not undergo surgery. Anatomopathological examination confirmed Conn’s adenoma in 70.4% (n = 10) and bilateral adrenal hyperplasia in 29.6% of cases (n = 4). Surgery led to normalization of blood pressure in only 50% of cases (n = 7) and a reduction in the number of antihypertensive treatments in 28.6% of cases (n = 4).

Given these results, we tried to determine the predicting factors related to this persistent hypertension. The patients were divided into two groups: Group A with a complete resolution of hypertension and group B with persistent hypertension.

Patients over the age of 50 years and with a longer duration of hypertension (> 5 years) were more likely to retain hypertension. We showed that a BMI < 30 kg/m^2^, lower SBP (< 140 mmHg), lower DBP (< 90 mmHg), increased GFR (> 90 mL/min/1.73 m^2^), low incidence of dyslipidemia, and low incidence of diabetes were predictive factors for normalization of hypertension after adrenalectomy. While we did not find a significant result for gender and the number of antihypertensive drugs used (Table 3). 

**Table 3.** Comparison of Predictive Factors in Patients with Resolution and Persistent Hypertension After Surgery

Variables	Resolution of Hypertension (n = 7)	Persistent Hypertension (n = 7)	Odds Ratio	IC 95%	P-Value
**Age (y)**	46.6 ± 12.1	52.0 ± 7.91	1.03	(1.0 - 1.06)	0.02
**Duration of hypertension for more than 5 years**	42.8	57.1	1.13	(1.07 - 1.19)	0.04
**Gender, female**	100	71.4	1.2	(1.06 - 1.4)	0.82
**Number of antihypertensive drugs **	2.70 ± 0.81	1.86 ± 0.9	1	(0.09 - 11.02)	1
**BMI (kg/m** ^**2**^ **)**	27.5 ± 3.32	29.8 ± 2.83	0.0154	(0.0005 - 0.44)	0.01
**SBP (mmhg)**	134 ± 21.1	142.8 ± 29.8	0.0667	(0.0046 - 0.97)	0.04
**DBP (mmhg)**	82.1 ± 12.8	93.5 ± 18.4	0.0667	(0.0046 - 0.97)	0.04
**eGFR (mL/min/1.73 m** ^**2**^ **)**	83.3 ± 11.4	78.2 ± 14	0.0303	(0.0012 - 0.76)	0.03
**Dyslipidemia**	28.6	57.1	0.0667	(0.0046 - 0.97)	0.04
**Diabetes**	42.8	57.1	0.0667	(0.0046 - 0.97)	0.04
**LVH**	28.5	57.14	3.33	(0.0046 - 0.97)	0.04
**Hypokalemia**	85.7	85.7	1	(0.36 - 30)	0.28
**Microalbuminuria**	0	57.1	19.28	(0.797 - 466.2)	0.06

Abbreviations: BMI, Body Mass Index; SBP, systolic blood pressure; DBP, diastolic blood pressure; LVH, left ventricular hypertrophy.

## 5. Discussion

In this current study, PHA represented 38.57% of all the etiologies and 84.37% of cases treated for hyperaldosteronism, which is a higher number than in the PAPY study (5). The percentage of female cases is consistent with the 64% female preponderance reported in the study by Kilani et al. (9). The mean age at diagnosis also aligns with previous findings (11). Hypertension and hypokalemia represented 66.66% of all modes of revelation, a finding that resembles what is provided in the literature (Table 4). The mean BMI value was very close to the values of other series, with 23.8 kg/m^2^ in the series by Kim et al. (13) and 24.3 kg/m^2^ in the series by Ishidoya et al. (14). The average hypertension onset age is similar to the results found in the literature (9, 11, 15). The percentage of patients with severe initial arterial hypertension (AH) is close to the PAPY study, where the prevalence of PHA increased from 6.6% for grade 1, to 15.5% for grade 2, and 19% for grade 3 hypertension.

**Table 4.** Comparison of Frequencies of Resistant Hypertension and Hypokalemia in the Literature

Variables	Severe and resistant hypertension (%)	Hypokalemia (%)
**Present Study**	37	67
**Kilani et al. (** 9 **)**	30	64
**Haddam et al. (** 10 **)**	65	57
**Cordoliani et al. (** 11 **)**	66	82

Concerning the etiological investigation, Lee et al. recently published a multicenter study in 2021 reporting that adrenal CT had an overall precision of 64.4% (16) (Table 2). The fact that CT scans are not always revealing is confirmed by our study. However, adrenal venous sampling (AVS) has long been considered the reference test (17, 18); unfortunately, it is not available in our context. Regarding complications, many studies have demonstrated that left ventricular hypertrophy (LVH) is more pronounced in patients with PHA and indicates a higher level of cardiovascular risk (19). Several studies have shown a link between PHA and metabolic syndrome, but causality has not been demonstrated. A meta-analysis including 4031 subjects in 16 studies reported a prevalence of diabetes of 15.22% in patients with PHA, very close to the prevalence of 16% in the cohort of Chen et al. (20). In terms of renal function, analysis of 46 studies comparing renal function in patients with PHA and those with essential hypertension showed a higher GFR in PHA of 3.37 mL/min and more severe albuminuria (20).

As for medical treatment, our adoption of spironolactone remains relatively low compared with the study by Cordoliani et al. (11), which was of the order of 35%, while that of Naem (21) was higher at 74%. An analysis of eleven studies of patients with hypertension and Conn’s adenoma surgically treated showed an overall cure rate for hypertension in the range of 41% to 51%. In cases where hypertension was not cured by surgery, seven of the eleven studies reported an improvement in blood pressure without cure (22).

Analysis of the risk factors behind the persistence of hypertension in our center found that advanced age and patients with a longer duration of hypertension maintained hypertension after surgery. There was no significant result for gender and the number of antihypertensive drugs used, while a BMI < 30 kg/m^2^, lower systolic BP (< 140 mmHg), lower diastolic BP (< 90 mmHg), increased GFR (> 90 mL/min/1.73 m^2^), low incidence of dyslipidemia, and low incidence of diabetes were predictive factors for normalization of hypertension after adrenalectomy. Some of our findings are consistent with the research data collected from a multicenter study conducted on 353 patients treated surgically for unilateral PHA, based on the PASO criteria. The study showed that BMI ≥ 25 kg/m^2^, male gender, advanced age, diabetes, long duration of hypertension, and decreased GFR were all related to persistent hypertension after surgery (23). Similarly, Zarnegar et al. concluded to a score based on 4 variables known preoperatively (number of antihypertensives ≤ 2, BMI ≤ 25 kg/m^2^, duration of hypertension ≥ 6 years, female gender) with a total score out of 5 points. A score of 0 or 1 point has a negative predictive value of 72.4%, while a score of 4 or 5 has a positive predictive value of 75% for the cure of hypertension (24).

It should be noted that our study has certain limitations, including the risk of missed follow-up and missing data, small sample size, and potential selection bias. Variability in pre- and postoperative management may also have impacted outcomes, as well as patient postoperative behavior. However, we believe that our results can enrich research in this area. As this is a monocentric study with a limited number of patients, the generalizability of the findings to broader populations should be approached with caution.

### 5.1. Conclusions

Primary aldosteronism (PA) often leads to resistant hypertension, highlighting the need for more studies in this area. It is necessary to establish a reliable predictive model to forecast the persistence of hypertension postoperatively. This will help strengthen the follow-up of the high-risk population, especially in regions like ours where research is still underdeveloped.

## Data Availability

The dataset analyzed during the present study is available from the corresponding author on reasonable request. However, the data are not publicly available due to ethical restrictions and patient confidentiality, in accordance with institutional and regulatory guidelines.

## Clinical Trial Registration Code:

Research registry 7745.
